# Small effective size limits performance in a novel environment

**DOI:** 10.1111/eva.12068

**Published:** 2013-04-03

**Authors:** Christopher G. Oakley

**Affiliations:** ^1^ Department of Biological Science Florida State University Tallahassee FL USA

**Keywords:** human disturbance, *Hypericum*, invasion, mutational meltdown, novel environment, roadside, small population

## Abstract

Understanding what limits or facilitates species' responses to human‐induced habitat change can provide insight for the control of invasive species and the conservation of small populations, as well as an arena for studying adaptation to realistic novel environments. Small effective size of ancestral populations could limit the establishment in, or response to, a novel or altered habitat because of low genetic variation for ecologically important traits, and/or because small populations harbor fixed deleterious mutations. I estimated the fitness of individuals from populations of the endangered plant *Hypericum cumulicola*, of known census and effective size, transplanted into native scrub habitat and unpaved roadsides, which are a novel habitat for this species. I found a significant positive relationship between estimates of population size and mean fitness, but only in the novel roadside habitat. Fitness was more than 200% greater in the roadside habitat than the scrub, mostly due to increased fecundity. These results combined with previous estimates of heterosis in this species suggest that fixed deleterious mutations could contribute to lower fitness of field transplants from small populations in the novel environment.

## Introduction

Anthropogenic habitat modification and climate change are becoming increasingly common (Kareiva et al. 1993; Parmesan 2006). An understanding of the factors that facilitate or hinder successful establishment and/or persistence in the face of anthropogenic change can be used to address challenges associated with human‐induced environmental change, such as controlling biological invasions (Sax et al. 2007; Callaway and Maron 2008; Salamin et al. 2010; Hendry et al. 2011; Fauvergue et al. 2012). Additionally, investigating factors that limit or facilitate establishment in a novel environment may provide insight into basic questions about adaptation to novel environments under realistic scenarios (Gomulkiewicz and Houle 2009; Crispo et al. 2010; Dyer et al. 2010; Gomulkiewicz et al. 2010). One major limitation to persistence in the face of habitat change and/or fragmentation may be the genetic consequences of small population size (Lynch and Lande 1993; Lynch et al. 1995; Higgins and Lynch 2001; Leimu et al. 2006, Willi et al. 2006).

Small populations may suffer a decreased ability to persist in the face of environmental change, and an increased risk of extinction by many mechanisms. Probability of initial persistence or establishment may be lower because decreased genetic variation reduces the chance that genotypes suitable for the novel habitat are present in the population, and reduces the continued ability to respond to changing conditions (reviewed in Frankham 1995; Leimu et al. 2006; Willi et al. 2006). Another potential threat to small populations is the accumulation of deleterious mutations (Lynch et al. 1995; Higgins and Lynch 2001). Empirical demonstrations of heterosis in crosses between populations, and in particular between small populations, provides good evidence that the fixation of mildy deleterious, partly recessive mutations may be important (Paland and Schmid 2003; Willi and Fischer 2005; Busch 2006; Oakley and Winn 2012). Such mutations are stochastically fixed within populations by genetic drift and are masked in the hetrozygous state in between population crosses, resulting in heterosis.

One approach to investigating the potential effects of population size on population persistence has been to examine the correlation between population size and components of fitness. In their meta‐analysis based on data from 45 such studies in 34 different species, Leimu et al. (2006) report a significant positive correlation between population census size and fitness components. Because all but four of these studies are observational, it cannot be determined how habitat quality influences these correlations. Populations may both be small and have low fitness simply because they occupy marginal habitats. In a handful of studies (Oostermeijer et al. 1994; Ouborg and van Treuren 1995; Fischer and Matthies 1998), plants were grown in the greenhouse to isolate the effect of population size. One study measured fitness components in an outdoor common garden (Fischer et al. 2003), but not in the natural habitat. Such approaches addresses intrinsic differences in fitness components among populations, but cannot address differences in fitness components that depend on the ecological context. The species invloved in most of these studies are threatened or endangered, and experimental common gardens in the native habitat may not be possible due to logistical and ethical constraints, even though this is the most relevant approach for addressing the effects of population size on fitness.

One feature common to many of the studies of the relationship between population size and fitness is that population sizes have been recently reduced due to habitat loss and fragmentation (Leimu et al. 2006), but an alternative scenario is the exposure of historically small populations to a novel environment as the result of anthropogenic modification. Novel environments are often expected to be stressful (Orr and Unckless 2008; Gomulkiewicz and Houle 2009; Agrawal and Whitlock 2010; Gomulkiewicz et al. 2010), but novel conditions may sometimes support increased reproductive output. This might be the case where disturbance reduces competition and increases resource availability (MacDougall and Turkington 2005; Dyer et al. 2010). Individuals from larger ancestral populations may be better able to capitalize on freed‐up resources in a novel environment than individuals from small populations because they posses fewer fixed deleterious mutations. The effect of deleterious mutations in a novel versus a native environment will likely depend on how stressful the novel environment is relative the native environment, because the expression of deleterious mutations is often greater in more stressful environments (Armbruster and Reed 2005; Cheptou and Donohue 2010; Fox and Reed 2010).

I addressed the effect of population size on performance in common gardens in both native and novel habitats, for multiple populations of the federally endangered perennial plant *Hypericum cumulicola* (Small) P.B. Adams, which has recently colonized a novel roadside habitat. Naturally occurring *H. cumulicola* in roadside habitats have shorter life spans, earlier times to first reproduction, and greater annual fecundity than plants in their native scrub habitat (Quintana‐Ascencio et al. 2007), suggesting that the roadside habitat constitutes a more favorable habitat. I transplanted individuals from populations ranging in size from just 15 to more than 1000 individuals (Oakley and Winn 2012) into native scrub and novel roadside habitats to address several questions: Is population size associated with higher fitness in either the native scrub or novel roadside environment? Is the novel roadside habitat a more favorable environment as measured by cumulative fitness? What are the relative contributions of survival and reproduction to differences in cumulative fitness? Using previously published estimates of heterosis for this species, I also ask whether fixed deleterious mutations could underlie the relationship between population size and fitness.

## Materials and methods

### Study system

*Hypericum cumulicola* is a federally endangered, short‐lived (average lifespan ~2 years) perennial plant. This species is endemic to the patchy rosemary scrub of the southern Lake Wales ridge in Florida, where it is a specialist of open sandy gaps between the shrubs (Quintana‐Ascencio and Menges 1996). Reproductive *H. cumulicola* plants can produce one to several hundred fruits per plant. Fruits are dehiscent capsules, and plants produce an average of ~11 small seeds per fruit (Oakley 2011). *Hypericum cumulicola* is characterized by wide variation in census and effective population size, and extreme spatial population structure (Dolan et al. 1999; Oakley and Winn 2012). *Hypericum cumulicola* is self‐compatible, but pollinator exclusion experiments suggest that the rate of autogamous selfing in this species is only around 7% (Evans et al. 2003). Direct assessment of population outcrossing rates is difficult due to low genetic variability for neutral markers (Oakley and Winn 2012), but limited among population variation for floral traits associated with self‐fertilization suggests that there are not dramatic differences among populations in their mating system (Oakley, unpublished manuscript).

The small size of some *H. cumulicola* populations, the highly discrete nature of habitat patches, and the virtual absence of migration between populations even at the scale of hundreds of meters suggest that genetic drift will shape patterns of genetic variation important for fitness (Oakley and Winn 2012). Genetic drift has been implicated in the fixation of partly recessive deleterious mutations within small populations. In a greenhouse study, based on the progeny from controlled hand pollinations, small populations show an average decrease in mean fitness of 68% compared to large populations, and an average increase in fitness in between‐population crosses of 70% (i.e. strong heterosis) compared to within‐population crosses (Oakley and Winn 2012).

Recent anthropogenic modifications of *H. cumulicola* habitat provide an opportunity to combine the detailed knowledge about variation in population sizes, degree of isolation, and levels of fixed deleterious mutations, with information on population level potential to establish and persist in a novel environment. This species has recently colonized unpaved roadsides and fire lanes that were created in the past 50–100 years and intersect native habitat patches. Many invasive plant species thrive in roadside habitats (Tyser and Worley 1992; Flory and Clay 2006; Christen and Matlack 2009), but the quality of roadside habitat for native species is not well understood. In *H. cumulicola*, sparser shrub cover and lower heterospecific root density (C.G. Oakley, unpublished data) suggest reduced interspecific competition in the novel roadside habitat compared to the native scrub environment. Comparative demography has shown dramatic differences in life history of plants in replicate natural scrub and road populations, with plants in the roadsides having 18% shorter lifespan, 23% earlier times to first reproduction, and up to 5‐ to 10‐fold higher annual fecundity than plants in the scrub (Quintana‐Ascencio et al. 2007), supporting consistent environmental differences between these two habitat types. Despite these differences, there is no evidence of local adaptation to these two habitat types (Oakley 2011).

### Population sampling and seedling generation

To determine the effect of population size on fitness in a native and a novel environment, I planted individuals from 15 populations ranging in census size from 15 to over 1000 individuals into each environment. In the fall of 2007, I collected open pollinated seed from each of 15 of the native scrub populations described in Oakley and Winn (2012). Seed collections were pooled from an average of 9.7 (range 6–12) maternal plants per population. For 14 of these populations, estimates of effective population size (mutation scaled inbreeding effective population size; 4N_e_μ, Table S1; see methods in Oakley and Winn 2012) calculated from microsatellite marker data using Migrate‐n (Beerli and Felsenstein 1999, 2001) are available from a prior study. These estimates are long‐term measures of effective population size that explicitly take into account migration between populations and are thus the best available measures of relative (putatively neutral) genetic diversity in these populations across long temporal scales (see also Charlesworth 2009).

In December 2008, I sowed a total of 5400 seeds (approximately 360 per population) in Petri dishes filled with field collected soil in an incubator set for 11 h days with 22°C days and 10°C nights. Once at least six seedlings had emerged for a population, I transplanted 3 per cell into randomized locations in seedling trays (5.7 cm diameter, 12.7 cm deep cells) filled with field‐collected soil. As seedlings emerged, I transplanted an average of 40 cells (2 per tray, 120 seedlings total) per population. I watered plants twice daily for 6 weeks with distilled water, and a 10% solution of 20‐20‐20 fertilizer was applied once after 4 weeks.

### Field transplant experiment

In March 2009, I transplanted 6‐week‐old seedlings into one scrub and one roadside common garden at Archbold Biological Station, near Lake Placid, FL. Less than 1% seed germination in the field (C.G. Oakley, unpublished data) precluded directly sowing seeds. In each habitat, I planted 10 blocks (seedling trays) of 30 cells containing ~3 seedling each at 10 cm spacing. Plants and intact root columns from individual cells were completely removed from their containers and planted directly into the sand. Blocks were placed randomly into available space with the exception that in the scrub, blocks were placed at least 0.5 m away from *Ceratiola ericoides* shrubs to prevent reduced sample sizes due to high early mortality (Quintana‐Ascencio et al. 1998). In all, I planted 290 cells in the scrub and 282 cells in the road, with an average of about 20 cells from each population in each habitat. Blocks were watered weekly for the first month to allow transplants to establish and covered with wire mesh cages during this time to prevent disturbance by animals attracted to the wet areas. After 2 weeks in the field to allow for mortality due to transplant shock, I randomly thinned seedlings to 1 per cell.

I recorded transplant survival to reproduction (only 2%, or 14 transplants survived to the end of the experiment but did not reproduce; survival to reproduction will hereafter refer to the combined probability of survival and reproduction), and harvested reproductive stems in mid‐October 2009 to determine the number of flowers and fruits produced. Because some individuals were still flowering at the time of harvest, I estimated total fruit production per plant as the number of fruits plus the product of the number of flowers and buds at time of harvest and the per plant proportion fruit set. For the second year, I scored survival to reproduction as above and harvested reproductive stems in mid‐November 2010. Because greater than 99% of plants had finished flowering, I counted total fruit production directly in this year. I quantified two fitness components: survival to reproduction over 2 years and cumulative fecundity (total number of fruits from individuals that reproduced, approximately 47 000 fruits counted experiment‐wide). Counts of seed number per fruit were impractical for this experiment. An estimate of cumulative fitness was calculated for each individual as the total estimated fruit production by the end of the experiment, including zeros for plants that died or failed to reproduce.

### Statistical analysis

#### Fitness in native and novel environments

As is common with estimates of fitness, the distribution of cumulative fitness in both environments is highly non‐normal due to an excess of zero or near zero values (Figure S1). I analyzed cumulative fitness over the course of the 2‐year experiment with a generalized linear mixed model anova (Proc GLIMMIX, SAS Institute 2008) with a Poisson error distribution and the canonical log link function (McCullagh and Nelder 1989). The Poisson error distribution model provided a much better fit to the data than a normal error distribution model (χ^2^/df = 63.17 vs 8607.43 respectively). This model included the effect of planting habitat as a fixed effect, and source population, the interaction of source population and planting habitat, and block nested within planting habitat as random effects. A significant effect of planting habitat would indicate environmental differences in mean fitness between the two habitats. A significant effect of population would indicate genetic differentiation among populations, and the interaction between planting habitat and population would indicate genetic differences among populations in their performance in the two different habitats. In the case of a significant interaction between population and planting habitat, models including the effects of population and block were also analyzed separately by planting habitat.

To provide additional support that the cumulative fitness results are not influenced by violations of distributional assumptions, I also analyzed this variable with Aster models (Geyer et al. 2007; Shaw et al. 2008). Aster models provide an analysis of variance of the joint distributions of different fitness components, in this case survival (binomial) and fecundity (zero‐truncated Poisson). Aster models do not accommodate random effects, so for these analyses, I treated all terms as fixed effects, otherwise the full model is the same as above. An additional limitation of Aster models is that all tests of effects are based on full likelihood comparisons of sets of nested models. It was therefore not possible to test all terms in the full model. My approach was thus as follows: Test the effect of the interaction between planting habitat and population by comparing the full model with a model with the interaction term removed, and if the interaction was significant, test for the effect of population within each planting habitat separately. For the purpose of significance testing, treating the interaction between population and planting habitat as a fixed effect versus a random effect should not matter because in either case, this term would be tested against the residual error variation. This is likewise true for the population effect in models run separately for each planting habitat. In either case, tests of the main effect of planting habitat with Aster models are less conservative because of the treatment of block and population as fixed effects.

In addition to cumulative fitness, I analyzed fitness components separately with generalized mixed model anova using the same model(s) as for cumulative fitness (above). Cumulative probability of survival to reproduction was analyzed with a binomial error distribution. Cumulative fecundity was analyzed with a Poisson error distribution.

#### Effect of population size on fitness

To address the effect of source population size on fitness of transplants in each environment, I examined the regression of population mean cumulative fitness on mutation scaled effective size. I also examined the regression with census number because no estimate of effective size was available for 1 of the 15 populations (Table S1; Oakley and Winn 2012). Census number and mutation scaled effective size estimates are correlated (*r *=* *0.82, *P *<* *0.001) for these populations. Regressions for fecundity were examined in the same way as for cumulative fitness. Heterogeneity of regression slopes for both cumulative fitness and fecundity was assessed by testing the interaction between either census or effective size and planting habitat. Because not enough seedlings germinated for all populations to be represented in every block, and because of strong variation among blocks (fivefold variation in block mean fitness in the roadside, and 42‐fold variation in block mean fitness in the scrub), population mean fitness was calculated from least square population means (treating population as a fixed effect for this purpose only because least square means cannot be calculated for random effects) separately for each planting habitat from models of the effects of block and population on cumulative fitness. Models with normal error distributions were used to facilitate interpretation of the least square means, but results are similar using least square means from models with Poisson error distributions (not shown).

## Results

### Fitness in native and novel environments

**Figure 1:**
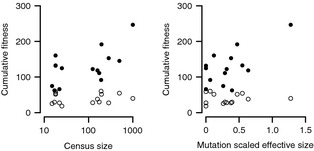
Population mean fitness in scrub and road planting habitats with respect to census size (left) and mutation scaled effective population size (right). Open circles represent plants grown in the scrub habitat, closed circles represent plants grown in the road habitat. Population means are least square population means to statistically remove the effect of block (see Fitness in native and novel environments).

Cumulative fitness over the 2 years of the experiment, including zeros for plants that died or failed to reproduce, was 229% greater in the roadside than in the native scrub (least square mean fruit number = 38 and 123 in the scrub and roadside respectively). This difference was significant (Table 1, Fig. 1). There was a significant interaction between population and planting habitat (Table 1, Fig. 1), indicating that the pattern of variation among populations differed in the two habitats. The main effect of population was not significant in the full model, but in models run separately for each planting habitat, the effect of population was significant in both the scrub (*Z* = 2.63, *P *=* *0.004) and the roadside (*Z* = 2.66, *P *=* *0.004) planting habitats. Similar results were obtained with the Aster framework: The interaction between population and planting habitat was highly significant (χ^2^ = 1239, df = 14, *P *<* *0.001) in the full model, and population terms were significant in separate models by planting habitat (χ^2^ = 1757, df = 14, *P *<* *0.001 and χ^2^ = 986.7, df = 14, *P *<* *0.001 in the roadside and scrub, respectively). Concordance between the results of Aster and more traditional anova models suggests that the generalized mixed model anova results are robust to violations of distributional assumptions.

**Table 1:** Generalized mixed model anova results for the effects of planting habitat (road and scrub), population, their interaction, and block nested within planting habitat on cumulative fitness. Population, the interaction between population and planting habitat, and block are treated as random effects

anova effects	df	*Z*	*F*	*P*
Planting habitat	1,14		12.63	0.003
Population		1.31		0.095
Population*Planting habitat		2.62		0.004
Block (Planting habitat)		3.15		<0.001

### Effect of population size on fitness

Estimates of population mean fitness in the novel roadside habitat were significantly positively associated with source population census size (*P *=* *0.002, *R* ^2^ **= 0.53) and mutation scaled effective size (*P *=* *0.012, *R* ^2^ **= 0.42):(Fig. 1, Table S1). In contrast, mean population cumulative fitness in the native scrub habitat was not significantly associated with source population census size (*P *=* *0.652, *R* ^2^ **= 0.02), or source population mutation scaled effective size (*P *=* *0.924, *R* ^2^ **= 0.00):(Fig. 1, Table S1). There was significant heterogenetity of slopes between planting habitats for both the regressions of fitness on census size (*P *=* *0.003), and on effective size (*P *=* *0.011), with steeper slopes in the roadside habitat (Fig. 1). Population mean fitness in the roadside habitat was not significantly correlated with population mean fitness in the scrub habitat (*r *=* *0.31, *P *=* *0.257).

### Components of fitness

**Figure 2:**
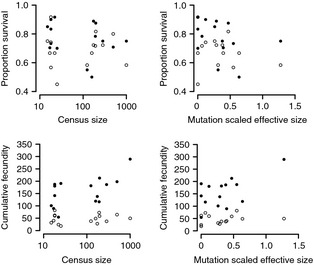
Population mean probability of survival to reproduction (top) and mean cumulative fecundity for plants that set fruit (bottom) in the native scrub environment (open circles) and the novel road environment (closed circles) plotted against population census size (left) and mutation scaled effective population size (right). Population mean probability of survival to reproduction values was calculated as the means of the block means. Mean fecundity values are least square means as in Figure 1.

The full model for cumulative probability of survival to reproduction failed to converge, but mean survival to reproduction was similar in the novel and native planting habitats (78% in the novel roadside vs 68% in the native scrub). In separate models by planting habitat, there was no significant population effect in either the scrub (*Z* = 0.56, *P *=* *0.287) or the roadside (*Z* = 0.49, *P *=* *0.313). Population mean probability of survival to reproduction ranged from 60% to 92% in the road and 45% to 92% in the scrub (Fig. 2). Cumulative fecundity was nearly three times greater in the roadside (mean fruit number = 164) than in the scrub (mean fruit number = 56) planting habitat (Fig. 2; *F*_1,14_ = 16.63, *P *=* *0.001). There was a significant interaction between planting habitat and population (*Z* = 2.61, *P *=* *0.005). Variation among populations in fecundity approached significance (*Z* = 1.47, *P *=* *0.071) in the full model, and population means ranged from 76 to 334 fruits in the road and 21 to 98 fruits in the scrub (Fig. 2). Separate analyses by planting habitat indicate significant differences among populations in both the scrub (Z = 2.61, *P *=* *0.005) and roadside (Z = 2.67, *P *=* *0.004) habitats. Regressions of fecundity with census and effective size (not shown) were qualitatively similar to the results for cumulative fitness.

### Relationship between estimates of heterosis and fitness in the field

Correlations between previously published estimates of heterosis (Oakley and Winn 2012) and the estimates of population mean cumulative fitness in the scrub and the road reported here are negative as expected, but non‐significant (*r *=* *−0.44, *P *=* *0.097, and *r *=* *−0.37, *P* = 0.178 for the road and scrub habitats respectively).

## Discussion

Recent construction of unpaved roads has created a novel habitat that has been colonized by the endangered plant *H. cumulicola*. I found that effective population size of natural scrub populations was significantly positively correlated with fitness of transplants to a novel roadside environment. I also found that fitness in the roadside was much greater than in the native scrub habitat and that this difference was largely attributable to greater fecundity in the roadside habitat.

Although there was differentiation among populations in cumulative fitness in both habitats (Fig. 1), population mean fitness was significantly correlated with population effective and census sizes only for plants grown in the novel roadside habitat. I am unaware of other studies that report both estimates of population size (census or effective) from natural populations and fitness in a natural environment. Newman and Pilson (1997) manipulated genetic variation, of experimental populations of the annual plant *Clarkia pulchella*, and found that populations with lower genetic variation had lower fitness and increased extinction rates in their native habitats. Because my experiment involved first generation progeny from field‐collected seed, environmental maternal effects (Roach and Wulff 1987) could have contributed to differences in population mean fitness. This seem unlikely as the major driver of the difference in the effect of population size in the two different environments, and I have found no evidence of environmental maternal effects in germination and seedling traits in this species (Schuster and Oakley, unpublished data).

The correlations between fitness in the novel environment and population size reported here (0.53 for census size, 0.42 for effective size) are similar to the overall average (~0.4) from observational studies, but greater than the average (~0.15) reported by studies using common gardens in more controlled conditions (Oostermeijer et al. 1994; Ouborg and van Treuren 1995; Fischer and Matthies 1998; Fischer et al. 2003; Leimu et al. 2006). One weakness of correlational studies is that differences in habitat quality could cause an association between population size and fitness independent of genetic causes, so comparisons of the results here with this body of literature must be made with caution.

The weak and non‐significant correlations between fitness in the native environment and population size are smaller than the averages for previous observational and common garden studies (reviewed in Leimu et al. 2006). One explanation that has been suggested for a lack of a significant correlation between genetic diversity (as a proxy for size) and fitness is high levels of gene flow (e.g. Leimu and Mutikainen 2005). This is not the case for *H. cumulicola*, as it has been demonstrated that most of these populations are strongly isolated (Oakley and Winn 2012). Extreme spatial heterogeneity at a fine scale could contribute to the weak correlation between population size and fitness in the native environment reported here. The 42‐fold variation in mean fitness among blocks that I found in the scrub habitat is likely a natural part of this species' environment, possibly driven by spatial variation in the distribution of competitors (Hunter and Menges 2002; Quintana‐Ascencio et al. 2007; Hewitt and Menges 2008). Regardless of its source, this extreme within site variation would make it difficult to detect any effect of population size.

I found over 200% greater fitness for plants growing in the novel roadside habitat than in the native scrub habitat. The difference was mainly due to greater fecundity in the roadside habitat (Fig. 2), rather than differences in survival, confirming consistent phenotypic differences in fecundity among replicate sites of each habitat reported for natural populations (Quintana‐Ascencio et al. 2007). Given that field germination rates are very low (Oakley, unpublished data, E. S. Menges, Pers. Comm.), high fecundity may be important for population establishment in the roadsides. Novel environmental conditions are often expected to be stressful (Orr and Unckless 2008; Gomulkiewicz and Houle 2009; Agrawal and Whitlock 2010; Gomulkiewicz et al. 2010), but here the disturbance associated with the creation and maintenance of a road may provide a release from competitively superior dominant shrubs (Quintana‐Ascencio et al. 2007) and/or from the negative biochemical effects of those shrubs, namely *Ceratiola ericoides* (Hunter and Menges 2002; Hewitt and Menges 2008). Although I used only one planting site in each habitat, previous work has demonstrated consistent phenotypic differences between replicate, naturally occurring populations in scrub and roadside habitats (Quintana‐Ascencio et al. 2007), which supports consistent differences in the two kinds of environments.

One mechanism that could underlie the greater ability of large populations to capitalize on favorable environmental conditions in the novel roadside habitat is a lower frequency of fixed deleterious mutations. Strong heterosis in crosses between small populations of *H. cumulicola* supports the presence of such fixed deleterious mutations (Oakley and Winn 2012). However, the effect of deleterious mutations is expected to be stronger in more stressful environments (Armbruster and Reed 2005; Cheptou and Donohue 2010; Fox and Reed 2010), thus we would expect the magnitude of the depressive effect of fixed deleterious mutations to be stronger in the native scrub than in the more favorable novel roadside environment, which does not appear to be the case. Indeed, the lack of a relationship between population size and fitness in the native environment was unexpected given that one was detected in a greenhouse study using progeny from controlled crosses (Oakley and Winn 2012). This difference could be explained in part by greater power to detect differences in the roadside habitat due to greater variation in fitness (range of 184.2 fruits in the road and 41.9 fruits in the scrub), and by the large among block variation in the native habitat. It is also possible (though unlikely) that the deleterious effects of some mutations are only expressed in the novel environment. While strong selection and/or population level extinction in the native habitat may have removed some mutations that are deleterious in the native habitat, such conditionally neutral mutations could drift to high frequency.

Correlations between previously published estimates of heterosis and measures of cumulative fitness here were not significant in either environment, although both were negative as expected. On average, populations with greater heterosis, indicating more fixed deleterious mutations, have lower fitness when planted in the field and this effect is somewhat stronger in the novel roadside habitat.

Because this experiment encompassed a single generation, lower genetic variation for traits useful in exploiting the better conditions in the roadside habitat is unlikely to have contributed to the greater fitness of transplants from larger populations. Furthermore, two lines of evidence suggest that a lack of phenotypic variation for ecologically important traits would not be sufficient to prevent small populations of *H. cumulicola* from capitalizing on better conditions in the novel roadside habitat. First, there are no significant differences in the strength or pattern of phenotypic selection on above‐ground growth or time to first flowering in the two habitats (Oakley 2011). Second, there are no significant relationships between coefficients of genetic variation and populations size for these traits, or for relative allocation to roots and shoots (C.G. Oakley, unpublished manuscript).

One caveat to the relationship between population size and fitness in the roadside habitat is that the very high fitness of the largest population has a strong influence on both the magnitude and significance of the regression (Fig. 1). I argue that the result for this population is not an artifact, but rather that the remaining populations are all small enough to suffer some consequences of small population size. *Hypericum cumulicola* is a narrowly endemic, federally endangered species, and few populations of large size exist. It may not be possible to increase the number of large populations, but inter‐population crosses could be conducted to generate populations with different levels of genetic diversity for future experimental studies. Such experiments could be implemented as part of restoration efforts that include demographic monitoring. The combination of experiments and management is a powerful approach for gaining information about basic questions while simultaneously refining current and future management practices (Latta 2008; Menges 2008).

Many species will face novel environments due to increased habitat loss, alteration, and fragmentation caused by human encroachment, as well as predicted changes in climate and/or sea level. Understanding what might constrain small populations from responding to these environmental changes provides an opportunity to examine how selection and drift interact to influence future adaptive evolution in natural populations (Salamin et al. 2010). My experiment does not address evolutionary response to a novel environment, but it does provide information about the relative likelihood of establishment in a novel environment, which is a prerequisite for any subsequent evolutionary response. This information may have practical application for understanding the genetic consequences of small population size for extinction risk of species of conservation concern (Lynch et al. 1995; Newman and Pilson 1997; Saccheri et al. 1998), particularly in a changing environment (Lynch and Lande 1993; Higgins and Lynch 2001; Gomulkiewicz and Houle 2009). My results show that small effective size is one factor that can limit establishment success in a novel environment and that fixed deleterious mutations may underlie this constraint.

## Acknowledgements

I am are grateful to A. A. Winn for her advice and support during all phases of this research. I am indebted to Archbold Biological Station, E. S. Menges, P. F. Quintana‐Ascencio, and C. W. Weekley for advice and logistical support. I thank J. Mola, J. Monge, M. Ojima, E. Peterson, M. G. Shuster, R. Walsh, and K. Wray for assistance with the work. Members of the D. W. Schemske laboratory and two anonymous reviewers provided useful feedback on versions of this manuscript. This project was funded by a Florida State University CRC grant to A. A. W. and an NSF Doctoral Dissertation Improvement Grant (DEB‐0808435) to A. A. W. and C. G. O.

## Data archiving statement

Data deposited in the Dryad repository: doi:10.5061/dryad.283g6.

## Supporting information


**Table S1.** Census size, mutation‐scaled effective population size estimates, estimates of H_s_ for 15 populations of *Hypericum cumulicola* (data from Oakley and Winn 2012), and least square mean (LSM) population fitness for the scrub and roadside habitats from the present study.

**Figure S1.** Distribution of individual cumulative fitness (total fruit number produced per plant including zeros) by planting habitat.
