# Supplementary material for: Small effective size limits performance in a novel environment
Source: Evol Appl. 2013 Apr 3;6(5):823–31. doi: 10.1111/eva.12068 (PMC5779127; doi:10.1111/eva.12068)
Supplement: Supplementary file 1 — Table S1. Census size, mutation‐scaled effective population size estimates, estimates of Hs for 15 populations of Hypericum cumulicola (data from Oakley and Winn 2012), and least square mean (LSM) population fitness for the scrub and roadside habitats from the present study. [file EVA-6-823-s001.docx]

Table S1. Census size and mutation-scaled effective population size estimates for 15 populations of *Hypericum cumulicola* (data from Oakley and Winn 2012) and least square mean (LSM) population fitnesses for the scrub and roadside habitats from the present study.

| Population | Census size | 4N_e_μ (95% credible interval) | Nei's gene diversity | LSM fitness in scrub | LSM fitness in road |
| --- | --- | --- | --- | --- | --- |
| 3 | 15 | 0.261 (0.024–0.508) | 0.014 | 25.37 | 74.73 |
| 4 | 17 | 0.393 (0.124–0.692) | 0.146 | 29.52 | 62.46 |
| 1 | 18 | 0.549 (0.264–0.868) | 0.204 | 54.27 | 132.57 |
| 6 | 18 | 0.001 (0–0.284) | 0 | 59.03 | 131.78 |
| 8 | 18 | 0.121 (0–0.336) | 0.236 | 51.48 | 160.44 |
| 5 | 22 | 0.001 (0–0.232) | 0.065 | 27.44 | 65.95 |
| 2 | 25 | 0.001 (0–0.280) | 0 | 18.37 | 124.73 |
| 16 | 124 | 0.313 (0.014–0.834) | 0.237 | 27.80 | 122.30 |
| 11 | 159 | 0.637 (0.332–0.988) | 0.183 | 37.95 | 118.59 |
| 10 | 174 | 0.287 (0.044–0.546) | 0.102 | 29.78 | 111.05 |
| 14 | 192 | 0.065 (0–0.326) | 0.005 | 60.27 | 91.68 |
| 12 | 196 | 0.471 (0.194–0.784) | 0.206 | 51.42 | 191.67 |
| 9 | 285 | 0.379 (0.116–0.666) | 0.151 | 27.35 | 152.91 |
| 13 | 497 | n/a | n/a | 54.66 | 145.26 |
| 15 | 1001 | 1.277 (0.884–1.750) | 0.237 | 39.86 | 246.70 |
