# Supplementary figures and images for: Small effective size limits performance in a novel environment
Source: Evol Appl. 2013 Apr 3;6(5):823–31. doi: 10.1111/eva.12068 (PMC5779127; doi:10.1111/eva.12068)

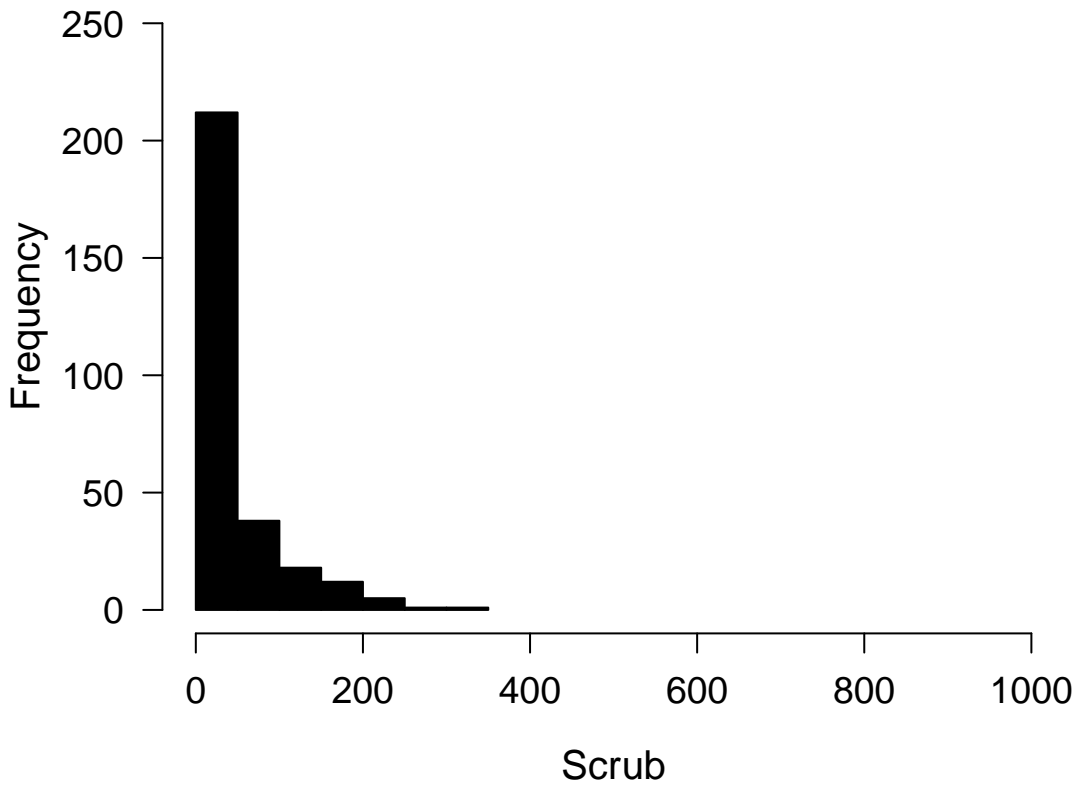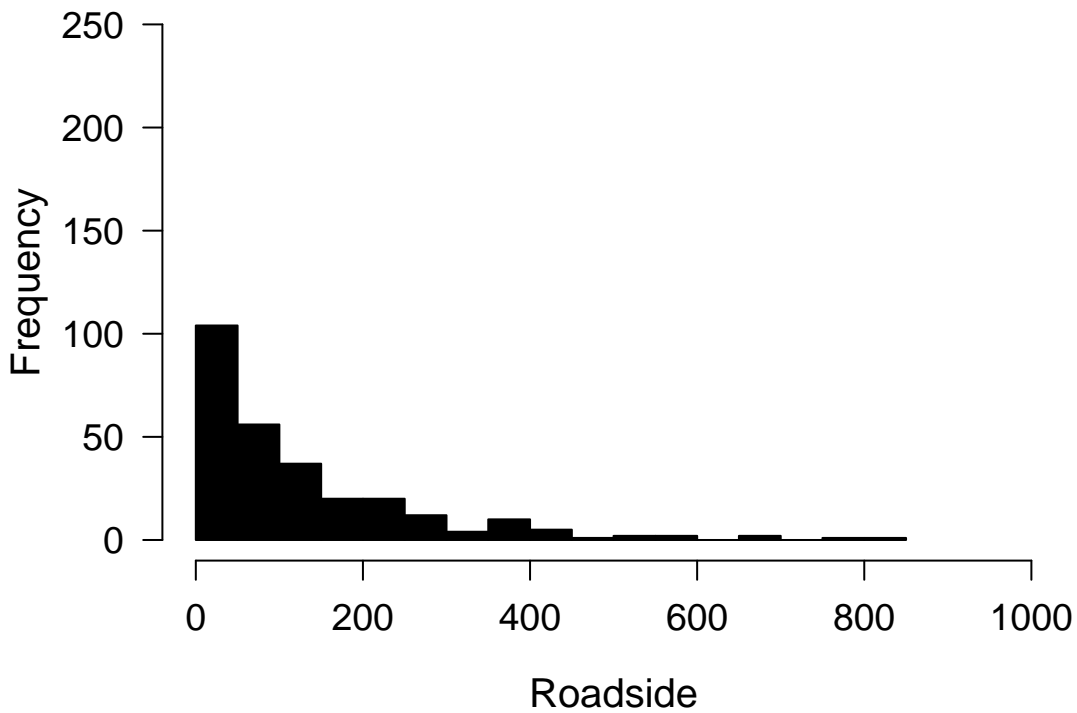

Supplement: Supplementary file 2 — Figure S1. Distribution of individual cumulative fitness (total fruit number produced per plant including zeros) by planting habitat. [file EVA-6-823-s002.pdf]
